# Effect of Membrane Hydrophobicity and Thickness on Energy-Efficient Dissolved Oxygen Removal From Algal Culture

**DOI:** 10.3389/fbioe.2020.00978

**Published:** 2020-08-21

**Authors:** Masatoshi Kishi, Kenta Nagatsuka, Tatsuki Toda

**Affiliations:** ^1^Faculty of Science and Engineering, Soka University, Tokyo, Japan; ^2^Plankton Eco-Engineering Research Center, Soka University, Tokyo, Japan; ^3^Graduate School of Science and Engineering, Soka University, Tokyo, Japan

**Keywords:** dissolved oxygen, gas-permeating, hydrophobicity, microporous membrane, thickness, pervaporation

## Abstract

Removal of dissolved oxygen from algal photobioreactors is essential for high productivity in mass cultivation. Gas-permeating photobioreactor that uses hydrophobic membranes to permeate dissolved oxygen (pervaporation) from its body itself is an energy-efficient option for oxygen removal. This study comparably evaluated the characteristics of various commercial membranes and determined the criteria for the selection of suitable ones for the gas-permeating photobioreactors. It was found that oxygen permeability is limited not by that in the membrane but in the liquid boundary layer. Membrane thickness had a negative effect on membrane oxygen permeability, but the effect was as minor as less than 3% compared with the liquid boundary layer. Due to this characteristic, the lamination of non-woven fabric with the microporous film did not significantly decrease the overall oxygen transfer coefficient. The permeability in the liquid boundary layer had a significantly positive relationship with the hydrophobicity. The highest overall oxygen transfer coefficients in the water-to-air and water-to-water oxygen removal tests were 2.1 ± 0.03 × 10^–5^ and 1.39 ± 0.09 × 10^–5^ m s^–1^, respectively. These values were considered effective in the dissolved oxygen removal from high-density algal culture to prevent oxygen inhibition. Furthermore, hydrophobicity was found to have a significant relationship also with water entry pressure, which needs to be high to avoid culture liquid leakage. Therefore, these results suggested that a microporous membrane with strong hydrophobicity laminated with non-woven fabric would be suitable characteristics for gas-permeating photobioreactor.

## Introduction

Microalgal mass-cultivation techniques for the production of pharmaceuticals, cosmetics, nutritional supplements, biomaterials, and biofuels have been widely studied over the decades (Burlew, 1953; Behera et al., 2015; Woortman et al., 2020). For the improvement of algal productivity, various components of algal cultivation have been intensively studied, including control and optimization of pH, culture medium, temperature, nutrient supply, and reactor design (Yew et al., 2019). While the high-density microalgal culture in a closed reactor is advantageous for high-quality biomass and low harvesting cost (Richmond., 2013), photosynthetic oxygen easily accumulates and inhibit the algal growth (Weissman et al., 1988; Kazbar et al., 2019). For example, the dissolved oxygen concentration exceeded 70 mg L^–1^ (more than 6 times the air saturation) in the outdoor *Arthrospira platensis* culture, at which the chlorophyll synthesis decreased to less than half (Torzillo et al., 1998). Prolonged exposure to high oxygen concentration can further lead to cell lysis (Vonshak et al., 1996). Especially in outdoor strong light conditions, the effect of oxygen inhibition increases owing to photorespiration (Shelp and Canvin, 1980; Molina et al., 2001). It is therefore necessary to reduce dissolved oxygen concentration. The most common and effective method of oxygen removal in a closed reactor is aeration. However, the aeration requires considerable high energy demand, and it may consist of 40% of the total cultivation and harvesting cost (Norsker et al., 2011). Low-cost oxygen removal is therefore a great concern in algal mass-cultivation.

**FIGURE 1:**
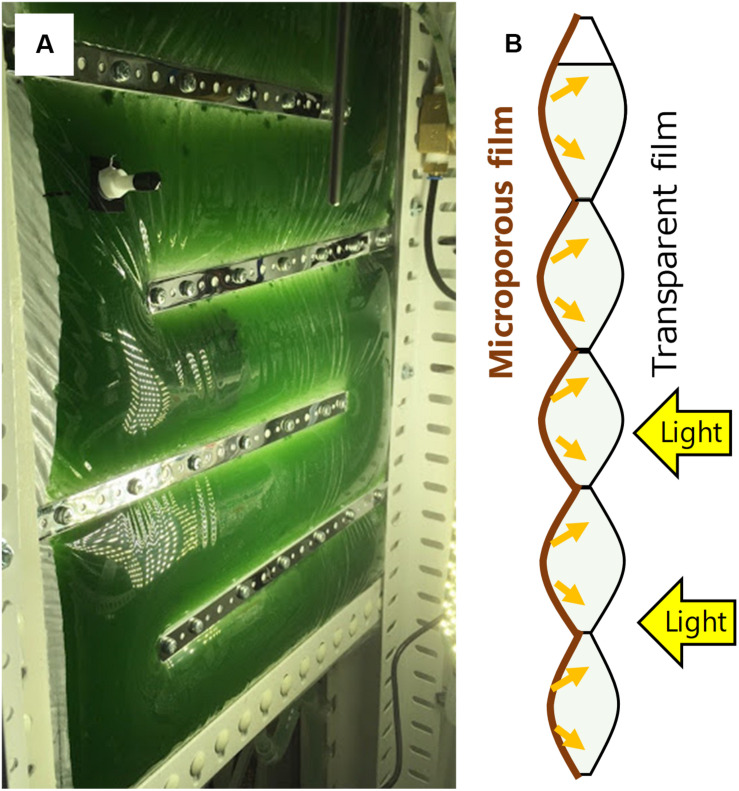
Gas-permeating algal photobioreactor **(A)** photo of the reactor in *Arthrospira platensis* culture; **(B)** Side view of a schematic diagram of the reactor [modified from Kishi (2018)]. Oxygen is removed from the microporous film side by diffusion. Light enters from the transparent film and reflects at the microporous film.

A recently developed concept of gas-permeating photobioreactor has demonstrated that a bag reactor partially made of a microporous film enables non-aerated dissolved oxygen removal by diffusion through the surface through pervaporation process (Kishi, 2018). This reactor was constructed by attaching a transparent plastic film and a microporous gas-permeating film, so that accumulated oxygen escapes to the atmosphere through the membrane without extensive aeration (Figure 1). Furthermore, the white membrane reflects the irradiated light inside the reactor enabling efficient light utilization. Under low mixing and aeration frequency, a significantly higher cyanobacterial growth was observed in the gas-permeating reactor compared with that in the conventional plastic bag reactor. This gas-permeating reactor may reduce the culture energy by 80 to 90% according to the results (Kishi, 2018) and can potentially be effective especially for high-value algal products that require closed conditions.

There have been many studies that used membranes for gas exchange in algal reactors such as for carbon dioxide supply (Lee and Hing, 1989; Pörs et al., 2010; Kim et al., 2011; Thrane et al., 2015; Mortezaeikia et al., 2016; Shesh et al., 2019). A recent floating bag reactor used gas-permeable membrane as a part of the sealing for oxygen release (Zhu et al., 2018b), but the membrane was meant to support the gas-gas exchange between the reactor headspace and the atmosphere. If the gas-permeating membrane is used as an interface between algal culture and the atmosphere (or water in the case of the submerged reactor), the specific surface of the membrane can be made large enough to remove the excess amount of oxygen via pervaporation. While gas-gas permeation is relatively simple because of negligible gas-boundary resistance, liquid-gas pervaporation is more complex since the liquid boundary layer often exhibit large resistance (Carvalho and Malcata, 2001). Oxygen removal through pervaporation has been attempted for semiconductors, beverages, and pharmaceuticals (Ito et al., 1998), but only a few studies have attempted dissolved oxygen removal from algal reactors through a membrane. Furthermore, most previous studies focused on the hollow-fiber membrane (Jana et al., 2017; Bazhenov et al., 2018; Su and Wei, 2019), while the gas-permeating photobioreactor utilizes a simple film as a part of the bag reactor surface that sustains the culture liquid. This structural difference creates differences in necessary criteria of membrane characteristics, namely tensile strength and water entry pressure, which is not necessarily important in hollow-fiber reactors. Thus, there is a need to characterize and select suitable membranes that have both high oxygen permeability and physical strength.

The membrane characteristics (e.g., material, pore size, thickness, and hydrophobicity) influence properties important in gas-permeating reactors, including oxygen permeability, physical strength (tensile strength and water entry pressure), and optical characteristics. Some characteristics such as thickness have both positive and negative effects; for instance, thicker membranes possess higher strength while having lower gas permeability. To select suitable membranes for the gas-permeating reactor, a comprehensive characterization of various properties is needed. Therefore, this study aimed to characterize different commercially available membranes and to reveal their effects on the properties of the gas-permeating reactor so that dissolved oxygen can be removed from a closed system without intensive energy.

## Materials and Methods

### Tested Films

The tested commercial films differed in materials (polyethylene [PE], polypropylene [PP], polytetrafluoroethylene [PTFE], or nylon), pore characteristics, attachment of non-woven fabric, and structure (stretched or woven) (Table 1). The films were categorized into four groups: standard microporous films (MP), a microporous film with non-woven fabric support (NW), woven porous film (WP), and non-porous films (NP). Silicone rubber film (NP-S) and polypropylene film (NP-P) were included as a comparison with the porous films. The thickness of NW films was measured using the scanning electron microscope. For the other films, the thickness value was derived from product information.

**TABLE 1:** Characteristics of the membranes used in this study.

ID	Material	Thickness (μm)	Pore size (μm)	Porosity (%)	Product name	Company	Model number
**Microporous film**							
MP1-1	PE	25	0.1	80	Miraim	Teijin	01–20
MP1-2	PE	30	0.2	80			02–35
MP1-3	PE	75	0.5	80			05–75
MP2	PP	38	0.3	21	Microporous Film	3M	–
MP3-1	PTFE	30	0.2	N.D.	Poreflon	Sumitomo	HP-020-30
MP3-2	PTFE	60	0.22	N.D.			FP-022-60
MP3-3	PTFE	80	0.2	N.D.			WP-020-80
**Microporous film with non-woven fabric support**							
NW1-1	PE	45*	N.D.	N.D.	Breathron	Nitto	BRN3000E1
	(Cloth: PET)	205*	N.D.	N.D.			
NW1-2	PE	50*	N.D.	N.D.			BRN-1860
	(Cloth: Nylon)	300*	N.D.	N.D.			
**Woven porous film**							
WP	Nylon	40	N.D.	N.D.	Silfine	Toyobo	EL1044PWR3
**Non-porous film**							
NP-S	Silicone	500	–	–	Silicone Rubber Sheet	Togawa Rubber	–
NP-PP	PP	50	–	–	Pylen	Toyobo	P8128

PE: polyethylene; PET: polyethylene terephthalate; PP: polypropylene; PTFE: polytetrafluoroethylene. N.D. no data; – not applicable; *Data measured with scanning electron microscopy. Other data were obtained from the product datasheet.

### Theory and Experimental Design of Oxygen Permeation

In gas permeation through the membrane, the permeability depends on the overall resistance (1/*K*) that is the sum of resistance at the membrane (1/*k*_M_) and in the fluid boundary layers on both sides of the membrane (1/*k*_A_ and 1/*k*_B_) (Carvalho and Meireles, 2006; Raisi and Aroujalian, 2011):

(1) $$\frac{1}{K}=\frac{1}{k_{\text{A}}}+\frac{1}{k_{\text{M}}}+\frac{1}{k_{\text{B}}}$$

where *K* is the overall transfer coefficient, *k*_*i*_ is the transfer coefficients for respective layers. For the case of gas to gas permeation, the gas diffusivity is much larger than that in the membrane, and thus oxygen permeability depends on the membrane (Yamamoto and Nakajima, 1979; Côté et al., 1989). Based on this assumption, Air-Air gas permeation was assumed to solely rely on membrane resistance, and Eq. 1 can be simplified to:

(2) $$\frac{1}{K_{\text{AA}}}=\frac{1}{k_{\text{M}}}$$

Similarly, in the case of Water-Air permeation, the resistance in the membrane and liquid boundary layer becomes the rate-limiting (Brüschke, 1990; Carvalho and Malcata, 2001; Raisi and Aroujalian, 2011). Thus, the overall resistance was assumed as the sum of the membrane and liquid boundary:

(3) $$\frac{1}{K_{\text{WA}}}=\frac{1}{k_{\text{M}}}+\frac{1}{k_{\text{B}}}$$

On another hand, Water–Water permeability requires consideration of resistance in all three layers:

(4) $$\frac{1}{K_{\text{WW}}}=\frac{1}{k_{\text{A}}}+\frac{1}{k_{\text{M}}}+\frac{1}{k_{\text{B}}}$$

In this estimation, *k*_M_ and *k*_B_ were assumed to remain constant throughout different permeation test conditions.

**FIGURE 2:**
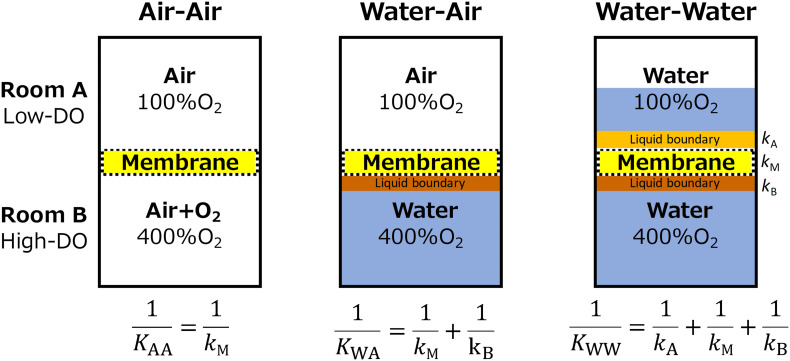
Design of oxygen permeability tests. Overall oxygen transfer coefficient (*K*) of each condition was used to deduce the transfer coefficient within the membrane (*k*_M_), upper liquid boundary (*k*_A_), and lower liquid boundary (*k*_B_). Transfer resistant between air and membrane was assumed to be negligible.

Upon the above presumptions, oxygen permeability of the membrane was evaluated under three conditions of Air-Air, Water-Air, and Water–Water phases (Figure 2). Oxygen permeability was measured using a hand-made acrylic device composed of two rooms (A and B). Room A was continuously flushed with water or air with an oxygen concentration of 100%air-saturation, while Room B contained high oxygen concentration (400%air-saturation) water or air. Using Eq. 1–3, the overall mass transfer coefficients (*K*) of each three conditions were used to estimate the transfer coefficient of (1) membrane (*k*_M_), (2) liquid boundary layer in Room B (*k*_B_), and (3) liquid boundary layer in Room A (*k*_A_).

The overall mass transfer coefficient *K* (m s^–1^) was estimated from the change of the dissolved oxygen concentration in Room B (*C*_B_; %air-saturation) using the following equation:

(5) $$\text{log}\,\frac{C_{\text{B}}\,\left(0\right)-C_{\text{A}}}{C_{\text{B}}\,\left(t\right)-C_{\text{A}}}=K\,a\,t$$

where *t* is the elapsed time (s); *C*_A_ is the dissolved oxygen concentration in Room A that was kept at 100%air-saturation; *a* is the specific surface of membrane over the volume of Room B (14.3 m^–1^).

### Analysis

Dissolved as well as gaseous oxygen concentration was measured using an optical oxygen sensor (FireStingO2, PyroScience) with contactless oxygen sensor spots (OXSP5, PyroScience). Film hydrophobicity was evaluated by a contact angle of 20 μL distilled water. Optical characteristics were measured using a spectrophotometer (UV-2450, Shimadzu) equipped with an integrating sphere. The absorption and reflection spectra of the wavelength between 400 and 700 nm were integrated to obtain total absorbance, transmittance, and reflectance of photosynthetically available radiations.

Tensile strength (N cm^–1^) was measured for MP3-1, NW1-2, WP, and NP-P using a material analyzer (A&D). The films were cut into 10 × 100 mm test strip for both horizontal and vertical direction. The tensile strength values for MP1 series and MP2 were obtained from their product datasheets. For standardization purposes, the more durable direction of the film was annotated as the vertical direction. Water entry pressure is the minimum pressure required to force water through the pores of a hydrophobic membrane, and its data was derived from product information for data analysis.

### Statistical Analysis

Linear regression analysis was used to determine relationships between membrane characteristics and functionality. All analyses were measured for at least three times except for the contact angle measurement, and average data was used for the regression analysis. *P* < 0.05 was considered significant.

## Results

### Oxygen Permeability Test

#### Air–Air Permeability

The Air-Air permeability test was implemented to estimate membrane permeability *k*_M_ (Eq. (2)). The lowest membrane permeability was observed with polypropylene film (NP-P), which was about 10^4^ smaller than microporous films (Table 2). Silicone rubber (NP-S) had nearly 5 times higher transfer coefficient than NP-P but was still a few magnitudes lower than microporous films. All microporous films had similar permeability despite different pore sizes, thickness, and material. Woven porous (WP) had significantly lower *k*_M_ than microporous films, and attachment of non-woven fabric support (NW) further decreased the membrane permeability. Within the NW products, the *k*_M_ of NW1-2 was approximately 5 times lower than NW1-1.

**TABLE 2:** Overall oxygen transfer coefficients and contact angle of various films.

Film ID	Film type	Air-Air permeation *K*_AA_ (×10^–^^3^ m s^–^^1^)	Water-Air permeation *K*_WA_ (×10^–^^5^ m s^–^^1^)	Water-Water permeation *K*_WW_ (×10^–^^5^ m s^–^^1^)	Contact angle (°)
MP1-1	Microporous; PE	2.00 ± 0.04	1.80 ± 0.13	1.26 ± 0.01	114
MP1-2	Microporous; PE	1.95 ± 0.04	1.90 ± 0.29	1.35 ± 0.04	122
MP1-3	Microporous; PE	1.90 ± 0.10	1.99 ± 0.06	1.39 ± 0.09	118
MP2	Microporous; PP	1.97 ± 0.01	1.96 ± 0.05	0.94 ± 0.06	100
MP3-1	Microporous; PTFE	2.01 ± 0.03	2.10 ± 0.03	1.16 ± 0.05	134
MP3-2	Microporous; PTFE	1.00 ± 0.02	2.00 ± 0.13	1.28 ± 0.10	116
MP3-3	Microporous; PTFE	0.21 ± 0.01	1.96 ± 0.12	1.34 ± 0.13	121
NW1-1	Nonwoven support	2.02 ± 0.01	1.76 ± 0.09	1.17 ± 0.04	110 [120]*
NW1-2	Nonwoven support	1.97 ± 0.06	1.70 ± 0.03	0.60 ± 0.03	108 [96]*
WP	Woven porous	1.67 ± 0.11	1.24 ± 0.14	0.43 ± 0.04	96
NP-S	Silicone	0.00075 ± 0.00008	1.16 ± 0.03	0.59 ± 0.04	94
NP-P	Dense PP film	0.00016 ± 0.00003	0.10 ± 0.04	0.05 ± 0.08	–
					

PE: polyethylene; PP: polypropylene; PTFE: polytetrafluoroethylene. *Contact angles for nonwoven fabric support for the NW series was shown in parenthesis.

**FIGURE 3:**
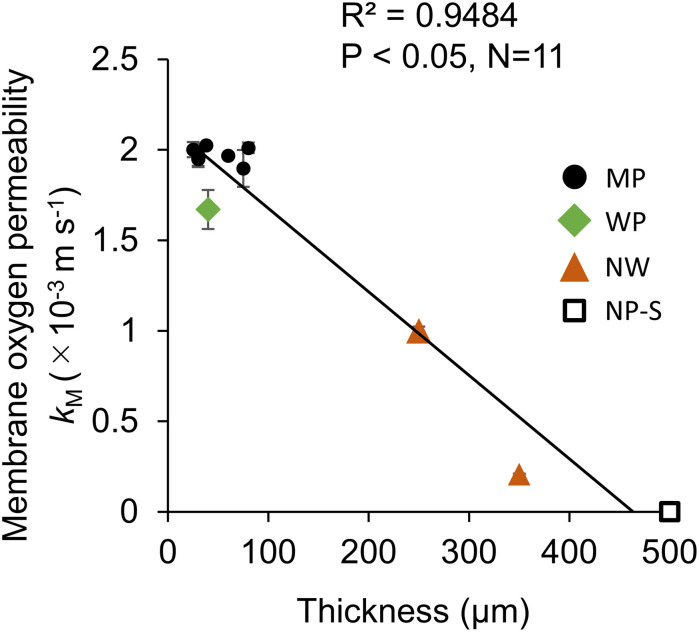
The relationship between oxygen transfer coefficient at the membrane (*k*_M_) and membrane thickness in the Air-Air permeation test. *N* = 3 for all the 11 films measured in this study. The error bar shows the standard deviation. MP: microporous; WP: woven porous; NW: microporous with a non-woven fabric; and NP-S: silicone films.

The membrane oxygen permeability *k*_M_ had a significant linear relationship with the thickness of permeable membranes excluding NP-P (Figure 3). Within the range of tested film, there was no correlation of *k*_M_ with pore size or porosity.

#### Water-Air Permeability

Compared with the Air-Air condition, the oxygen transfer coefficients decreased to nearly 1% (Table 2). While NW had lower transfer coefficients than MP series in Air-Air permeability, there was no significant difference among MP and NW series in Water-Air permeability. Compared to the other films, WP, NP-S, and NP-P were significantly lower. However, the difference between other films and NP-S became smaller compared to Air-Air permeation. The NP-S transfer coefficient increased from the Air-Air condition by 15 times.

#### Water–Water Permeability

The oxygen transfer coefficients were about half of the Water-Air permeability values for most of the films (Table 2). There was a clear difference in NW1-1 and NW1-2 in terms of reduction of *K;* although Water-Air transfer coefficients were comparable for the two, Water–Water permeability was lower by half for NW1-2.

**FIGURE 4:**
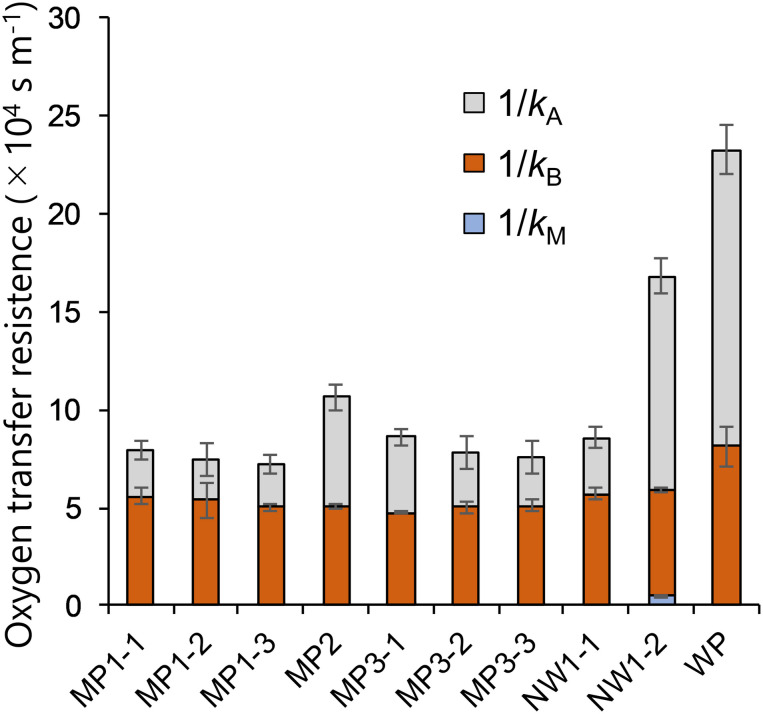
Breakdown of mass transfer resistance in the Water–Water oxygen permeation test. *N* = 3 for all membranes. Error bars show the standard deviation of each breakdown.

The oxygen transfer coefficients of Air-Air, Water-Air, and Water–Water permeability tests were used to derive relative contributions of *k_M_, k_B_*, and *k*_A_ on the overall resistance of Water–Water permeation test (Figure 4). Membrane resistance consisted of less than 1% of the total resistance except for the NW series, which was still less than 3%. Liquid boundaries in both sides of the membrane exhibited the major resistance in the permeability of oxygen. For NP-S and NP-P, due to the higher permeability in Water-Air and Water–Water conditions (in which combined resistance of membrane and both sides of the liquid boundary were assumed) compared to Air-Air conditions (in which only membrane resistance was assumed), the relative contributions were not calculated.

### Physicochemical Characteristics of Films and Their Effects

**FIGURE 5:**
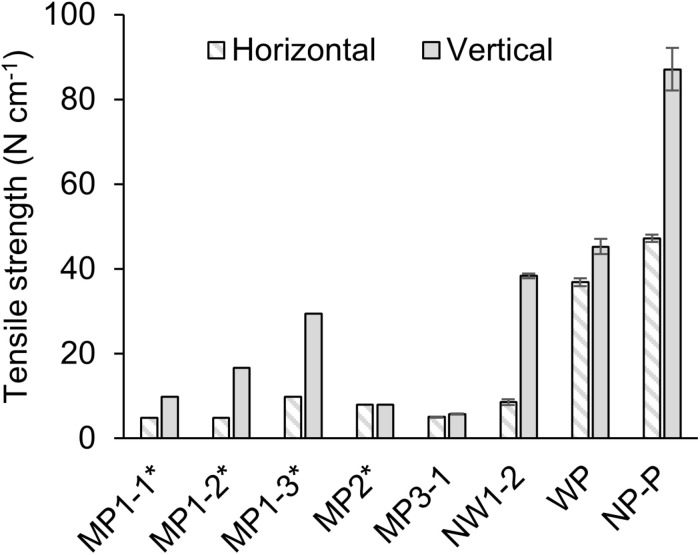
Tensile strength of the films. The error bar shows the standard deviation of *N* = 3. The values with asterisks (*) were derived from the product datasheet. MP: microporous; WP: woven porous; NW: microporous with a non-woven fabric; and NP-P: non-porous polypropylene film.

Tensile strength was measured for selected films (Figure 5). Dense polypropylene film (NP-P) had the highest strength, and the woven porous film was the next, showing about half of NP-P. Microporous films generally exhibited low strength although MP1 series had relatively stronger characteristics in one direction. Compared to the microporous film, attachment of non-woven fabric support improved the strength in one direction as much as woven film. There was no significant relationship between tensile strength and thickness or pore characteristics.

Contact angle that indicates hydrophobicity of films was in the range of 94 to 134°, indicating moderate to strong hydrophobicity (Table 2). PTFE and PET indicated relatively stronger hydrophobicity, while nylon was less water repellent.

**FIGURE 6:**
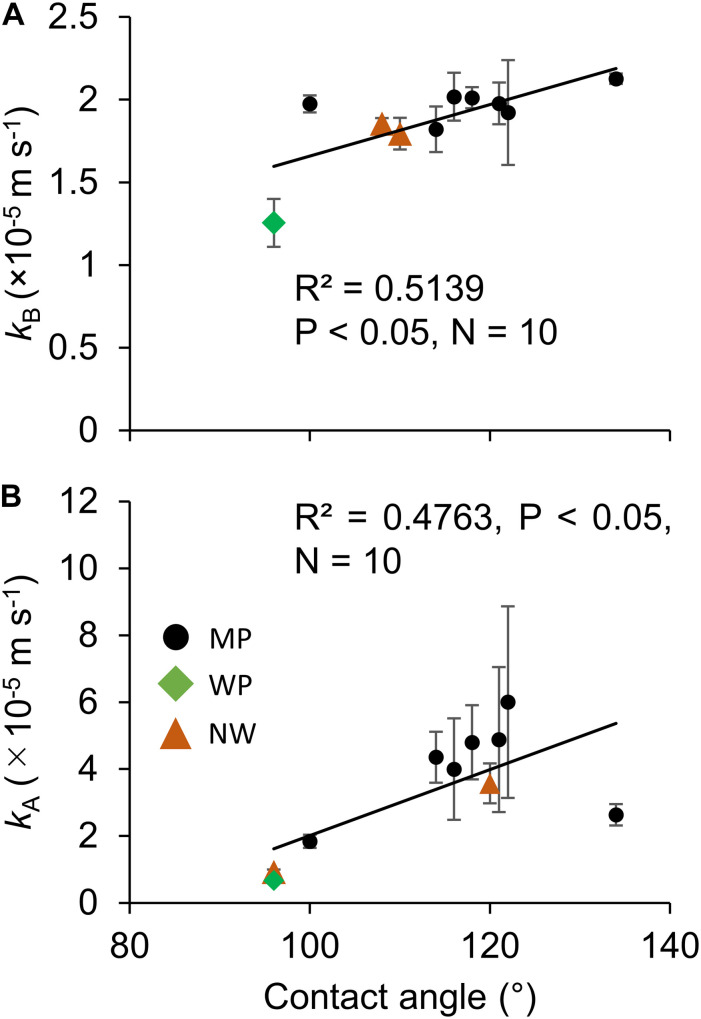
The effect of hydrophobicity (contact angle) on the oxygen transfer coefficients in **(A)** Room B and **(B)** Room A. *N* = 3 for all the 10 microporous membranes measured in this study. The error bar shows the standard deviation. MP: microporous; WP: woven porous; and NW: microporous with a non-woven fabric.

**FIGURE 7:**
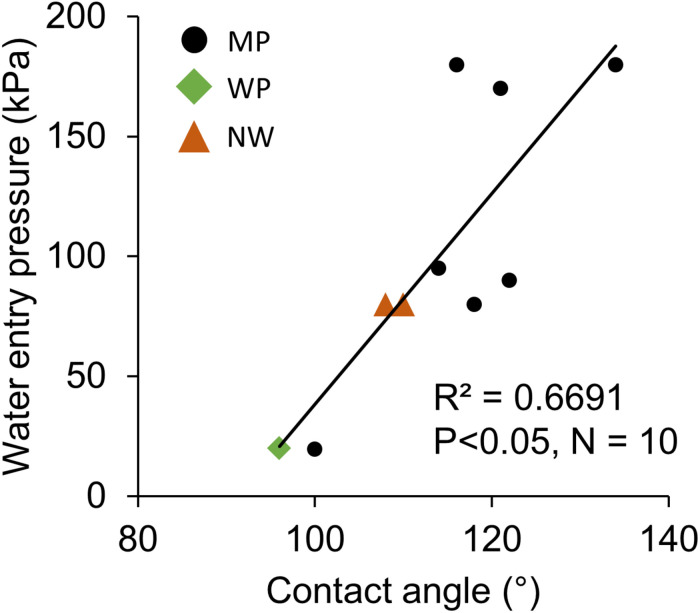
The positive relation between water entry pressure and hydrophobicity (contact angle). *N* = 1 for all the 10 microporous membranes measured in this study. MP: microporous; WP: woven porous; and NW: microporous with a non-woven fabric.

The contact angle exhibited significant correlations with oxygen permeability in both sides of liquid boundary layers, *k*_B_ and *k*_A_, across the various types of porous membranes (Figures 6A,B). Although transfer coefficients *k*_A_ was calculated by subtracting *k*_M_ and *k*_B_ from the overall coefficient of Water–Water permeation, a significant correlation was observed (Figure 6B). The effect of different non-woven fabric hydrophobicity was also taken into consideration for *k*_A_. The contact angle also had a positive correlation with water entry pressure (Figure 7).

**FIGURE 8:**
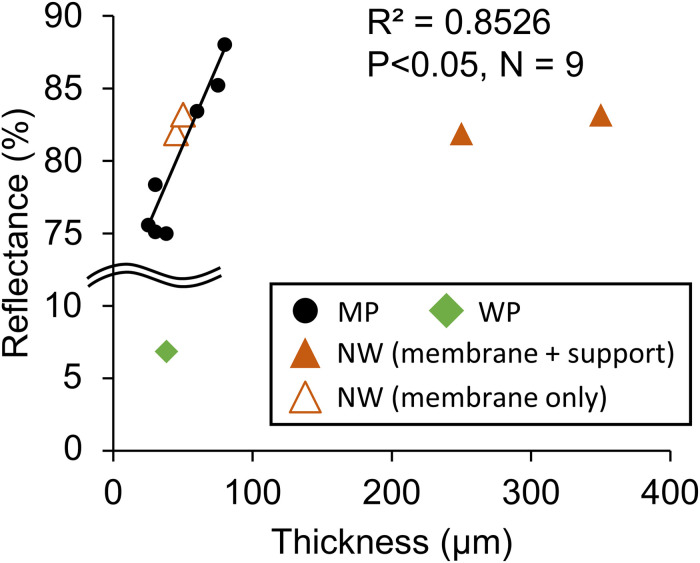
Relationship between optical reflectance and membrane thickness. MP: microporous; WP: woven porous; and NW: microporous with non-woven fabric support. The thickness of the membrane only was adopted for NW in the regression line. WP was removed from the regression analysis due to its black dye.

The optical characteristics of the tested membranes indicated that most porous membranes except WP, which is dyed with black paint, had high reflectance of approximately >80%. The reflectance rate of those white membranes correlated with the thickness (Figure 8). In the correlation analysis of NW, membrane thickness excluding non-woven fabric was applied.

## Discussion

### Oxygen Transfer

The membrane permeability had little effect on the dissolved oxygen removal as *k*_M_ was only less than 3% of the overall oxygen resistance from water because of much higher resistance in the liquid layers (Figure 4). The reason behind the smaller resistance could be the relatively large pore size and the hydrophobicity that may have filled the pore with gas. This finding was similar to those of CO_2_ supply (Carvalho and Malcata, 2001) in which the effect of membrane permeability was considered negligible when Henry’s constant was high. This low contribution in resistance was the case even with a membrane laminated with non-woven fabric support. Although the membrane gas transfer coefficient of NW1-2 was less than 10% of MP membranes (Table 2) due to the increased thickness (Figure 3), this resistance did not affect much on the overall oxygen transfer from water to air (Figure 4). This result was somewhat inconsistent with previous studies that stated increased modeling accuracy by the inclusion of the support layer in the estimation of pervaporation flux (Heintz and Stephan, 1994; Lipnizki et al., 2002). However, these studies dealt with a more accurate estimation of transfer using much smaller pore-size support (0.016 μm). In the rough estimation like this study, the liquid boundary transfer coefficient can be solely considered for dissolved oxygen removal. Thus, attachment of large-pore support like the non-woven fabric was found to be effective in increasing the membrane durability (Figure 5), while not negatively affecting dissolved oxygen removal properties.

Reduction in the liquid boundary transfer coefficient was found to be the most important factor in dissolved oxygen removal. The current study described that increasing hydrophobicity improves oxygen permeability (Figure 6), and the highest *k*_B_ of 2.13 × 10^–5^ m s^–1^ was achieved with MP3-1 of the highest hydrophobicity. This oxygen transfer coefficient was similar to a previous study of 1.2–2.5 × 10^–5^ m s^–1^ (Kishi, 2018), 0.052–0.77 × 10^–5^ m s^–1^ (Vladisavljević, 1999), 0.44–1.56 × 10^–5^ m s^–1^ (Su, 2018), 0.75–4.65 × 10^–5^ m s^–1^ (Bhaumik et al., 2004), and 1.1–3.4 × 10^–5^ m s^–1^ (Côté et al., 1989). This coefficient enables maintenance of dissolved oxygen at 200% with a gas-permeating reactor having 1 g L^–1^ d^–1^ of algal biomass productivity, based on the assumption of the gas-permeating bag reactor configuration (Kishi, 2018): specific membrane surface area of 87 m^–1^ and oxygen production to biomass production ratio of 1.1 g g^–1^. These results suggested that an energy-efficient oxygen removal from a closed reactor is possible through the use of a hydrophobic membrane to prevent oxygen inhibition without intensive aeration.

The overall oxygen transfer coefficients in the Water–Water permeation test were in the range of 0.43 to 1.39 × 10^–5^ m s^–1^ (Table 2). Although this is a comparably smaller value than Water-Air permeation, 1.39 × 10^–5^ m s^–1^ of oxygen transfer, for example, can maintain dissolved oxygen at 250% with the same assumption as above. Considering over 600% oxygen concentration in a closed photobioreactor (Torzillo et al., 1998), this oxygen concentration maintenance can be considered effective. Therefore, this result suggests that gas-permeating photobioreactors can be operated facing water such as that of the Solix system where whole bag reactors are submerged within a water bath for effective temperature control (Morweiser et al., 2010). Care needs to be taken in choosing appropriate film support with hydrophobicity, since liquid boundary layer on the low-oxygen side matters in Water–Water oxygen permeation.

Interestingly, silicone rubber had *K*_WA_ and *K*_WW_ close to those of porous films despite distinctively lower *K*_AA_ (≒*k*_M_), which was a few magnitudes lower than *K*_WA_ and *K*_WW_ (Table 2). The mechanism behind this characteristic is not clear as far as the authors’ knowledge, but a relatively hydrophilic feature of dense silicone may have supported faster diffusion of gas through the membrane with the existence of water than without water.

### Water Entry Pressure and Optical Properties

Water entry pressure was higher for increasingly hydrophobic films (Figure 7). The Young-Laplace equation states that water breakthrough pressure *P* (a similar index as the water entry pressure) (Scott and Barker, 2006) positively correlates with contact angle θ and inversely correlates with pore radius *r* (Di Felice, 2016; Bazhenov et al., 2018):

(6) $$P=\frac{-2\,\sigma \,c\,o\,s\,\theta }{r}$$

where σ is liquid surface tension. Although the relationship between equation-based estimation and actual water entry pressure did not correspond well (*R*^2^ = 0.26), the theory explains well that water repellency avoids water permeation through membrane pores. As a low water entry pressure leads to algal culture leakage through the membrane, a moderately high value is favorable. For example, solely considering the static pressure, water entry pressure of 20 kPa in MP2 can only sustain a 2-m water head, while 180 kPa in MP3-1 can sustain up to an 18-m water head. Considering the dynamic pressure due to culture mixing, a higher water entry pressure is demanded to prevent culture leakage.

Optical reflectance positively correlated with membrane thickness (Figure 8). The mechanisms for this correlation could be that the microstructure refracted light, and the thicker the film, the more potent light is backscattered. The reason for NW being out of the correlation could be because the non-woven fabric is much less dense than the microporous film, and light escaped through the large pores. Due to the high reflectance of most of the porous films, the gas-permeating photobioreactor was designed to utilize the reflectance of light (Kishi, 2018). Thus, the thicker film may be somewhat beneficial for the gas-permeating reactor, as an increase in membrane oxygen permeation does not deteriorate dissolved oxygen removal much.

### Suggested Gas-Permeating System for Algal Photobioreactor

**FIGURE 9:**
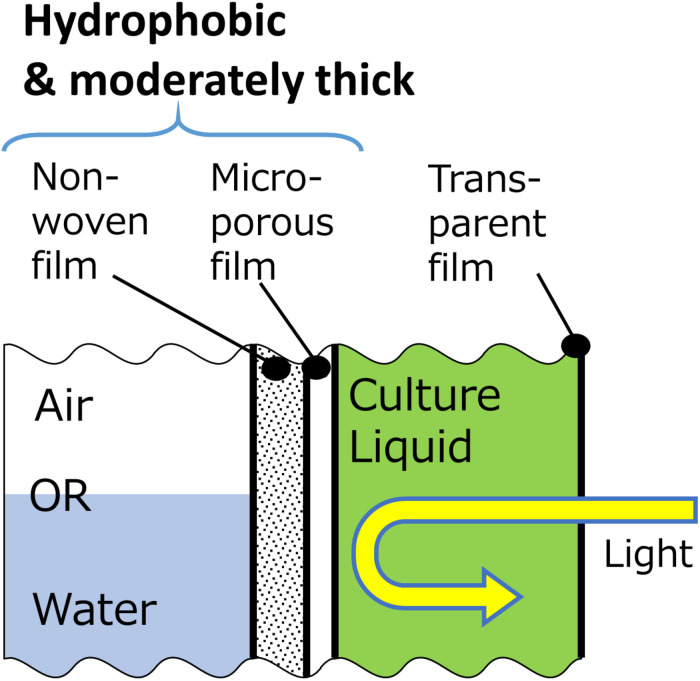
Proposed schematic structure of a gas-permeating photobioreactor composed of three film layers for dissolved oxygen removal.

The results of this study indicated that there are favorable physicochemical properties and structure of membranes that are suitable for the gas-permeating photobioreactor (Figure 9). Firstly, for the improvement of oxygen permeability, hydrophobicity of membranes facing water needs to be high. Secondly, since the thickness of the membrane does not affect the overall dissolved oxygen transfer, attachment of porous support and increase in membrane thickness would be beneficial to increase the strength and optical reflectance. Such characteristics and structure would enable a durable oxygen-permeating photobioreactor.

The suggested gas-permeating membrane system can be applied to various types of algal photobioreactors. A bag reactor supported by iron frames (Sierra et al., 2008), hanged column-type bag reactors (Abomohra et al., 2014), and multi-folded reactors (Schreiber et al., 2017) are largely operated examples of bag reactors. A gas-permeable membrane can be added to such reactors for energy-efficient dissolved oxygen removal without interfering the productivity, as long as the membrane does not shade the algal culture. The recent development in submerged and floating bag reactors can be interesting applications of the membrane dissolved oxygen system. For example, Solix proposed a completely submerged flat-panel bag photobioreactor system for better temperature control (Morweiser et al., 2010). Another example is floating bag reactors such as that utilizes wave for mixing the algal culture (Zhu et al., 2018a, b). Since Water–Water oxygen permeability of hydrophobic membrane was found to be high enough for maintaining dissolved oxygen concentration of algal culture at an adequately low level, the membrane can be used as a part of the culture container for those submerged and floating reactors. However, if the surrounding water is an uncontrolled environment like the ocean, biofouling on the outer side can be a serious issue, as reported in Zhu et al. (2018b).

For the practical selection of the membrane, however, additional cost and technical analysis are required. For instance, although the highest hydrophobicity was achieved with PTFE membranes, its cost is relatively high, and the construction of a bag reactor may be challenging due to its difficulty in attaching with other materials. Recent developments in film surface hydrophobization (Tuteja et al., 2007; Xue et al., 2009, 2014) may make cheap and easily handleable microporous films suitable for the gas-permeating photobioreactor.

In addition, further long-term cultivation tests are needed to evaluate the formation of microbial biofilm on the surface of the hydrophobic membrane, as the hydrophobic surface tends to easily develop microbial adhesion (Mazumder et al., 2010). Although long-term durability (ca. 100 days) of a microporous film in a gas-permeating reactor was indicated in a previous study with *Arthrospira platensis* culture (Kishi, 2018), the culture condition of extremely high alkalinity may have majorly contributed to this favorable results.

## Conclusion

This study compared the oxygen permeability of various films to clarify the suitable criteria for selecting membrane in an energy-efficient dissolved oxygen removal from algal reactors. The oxygen permeation test in Air-Air, Water-Air, and Water–Water conditions revealed the relative importance of liquid boundary layer resistance compared to the membrane resistance, which only consisted of less than 3% of the overall resistance. Membrane hydrophobicity was found to improve the liquid boundary oxygen transfer as well as water entry pressure. Moreover, since the oxygen transfer resistance in the membrane is neglectable, attachment of non-woven fabric support for improved strength was found to be a good option for gas-permeating bag reactor. Using the membrane with the highest hydrophobicity and oxygen permeability, the overall oxygen transfer coefficient was found to be high enough to maintain adequately low levels of dissolved oxygen in highly productive algal culture. Moreover, the overall oxygen transfer coefficients in both Water-Air and Water–Water conditions were high, indicating that the gas-permeating membrane can be also applied to floating and submerged conditions.

## Data Availability Statement

The raw data supporting the conclusions of this article will be made available by the authors, without undue reservation.

## Author Contributions

MK, KN, and TT contributed to the conception and design of the study. KN implemented most of the experimental works. MK performed the statistical analysis and wrote the first draft of the manuscript. All authors contributed to the manuscript revision, read, and approved the submitted version.

## Conflict of Interest

The authors declare that the research was conducted in the absence of any commercial or financial relationships that could be construed as a potential conflict of interest.

## Abbreviations

*k*_A_: oxygen transfer coefficient in the boundary layer in the Room A (low oxygen side) [m s^–1^]
*K*_AA_*K*_WA_*K*_WW_: overall oxygen transfer coefficient for Air-Air Water-Air, and Water–Water permeation conditions respectively [m s^–1^]
*k*_B_: oxygen transfer coefficient in the boundary layer in the Room B (high oxygen side) [m s^–1^]
*k*_M_: oxygen transfer coefficient in the membrane [m s^–1^]
MP: microporous films
NP: non-porous films
NW: microporous films with non-woven fabric support
PE: polyethylene
PP: polypropylene
PTFE: polytetrafluoroethylene
WP: woven porous films.
